# Fermentation of Youlk, an Australian Native Root Vegetable, Using Defined Lactic Acid Bacterial Strains

**DOI:** 10.3390/foods15111973

**Published:** 2026-06-02

**Authors:** Thilakna Ampemohotti, Aida Golneshin, Charles Brennan, Christopher Pillidge, Thi Thu Hao Van

**Affiliations:** 1 School of Science, RMIT University, Bundoora, VIC 3083, Australia; thilakna.ampemohotti@rmit.edu.au (T.A.); charles.brennan@rmit.edu.au (C.B.); christopher.pillidge@rmit.edu.au (C.P.); 2 Edlyn Foods Pty Ltd., Epping, VIC 3076, Australia; agolneshin@edlyn.com.au

**Keywords:** youlk, Australian native pepper, fermented food, lactic acid bacteria, volatile organic compounds

## Abstract

Youlk (*Platusace deflexa*) is a native root vegetable in Australia that is primarily consumed fresh, baked, or roasted. This study aimed to produce a fermented product using youlk with added lactic acid bacteria (LAB), in comparison with spontaneous fermentations. Three LAB strains were selected for their high in vitro antioxidant activity, leading to eight formulations that featured individual strains or a 1:1:1 combination. The strains were *Latilactobacillus sakei* F1, *Lacticaseibacillus paracasei* D2 and *Lacticaseibacillus rhamnosus* JL, all previously isolated from fermented vegetables. Two salt levels (2% and 4%) were used, together with the addition of 1% Australian native pepper (*Tasmannia lanceolata*). LAB counts, pH, titratable acidity, organic acid content, and volatile organic compounds (VOCs) were monitored over a three-week period. LAB viable numbers (7.5–9.3 log CFU/g), lactic acid content (1770–2740 ppm) and titratable acidity (0.72–0.89%) were significantly higher and pH was lower (<4.0 for the combined strains) in the LAB-inoculated group compared with the spontaneously fermented control group. The opportunistic pathogen *Pluralibacter gergoviae* was present in spontaneously fermented youlk but not in the LAB-inoculated group, likely due to lower pH. A total of 21 VOCs were detected, with α-pinene being the most abundant. Aroma-enhancing compounds like acetoin and linalool increased significantly by day 14. Principal component analysis (PCA) indicated that fermentation duration influenced VOC production more than the LAB strains. These results suggest that youlk is a promising candidate for fermented food production, with LAB strains enhancing fermentation and providing advantages over spontaneous fermentation.

## 1. Introduction

Youlk (*Platysace deflexa*), also known as Ravensthorpe radish or “bush carrot” by the indigenous Australian peoples, is a native root vegetable belonging to the Apiaceae (Umbelliferae) family. It is a tuberous perennial shrub that grows up to 0.6 m in height with small, green, ovate leaves, producing white, star-shaped flowers that bloom throughout the year. Youlk can be harvested year-round, but it is primarily harvested from October to December. It has a firm texture similar to that of an apple and has a golden, potato-like appearance, making it a desirable addition in food recipes. Its long shelf life under proper storage conditions is a major advantage [1]. It has commercial potential as a food, along with two other native root vegetables, *Ipomoea calobra* and *Haemodorum spicatum* [1]. In addition to these, native pepper berry or mountain pepper (*Tasmannia lanceolata*) has known food and medicinal benefits. In foods, it serves as a substitute for conventional pepper, adding herbal flavour and imparting a rich plum hue to sauces. It also has a long history of traditional use, and more recent scientific investigations have confirmed its medicinal properties, including antioxidant [2,3], anticancer [4], antidiabetic [5], and antimicrobial effects [6].

A large number of fermented vegetable food products (often with added health benefits) are available in the market, most of which have traditional origins. However, foods that have undergone spontaneous (natural) fermentations may be more prone to contamination by pathogenic bacteria and are more sensitive to changes in environmental conditions, affecting product consistency [7,8,9,10]. As a result, controlled or culture-dependent fermentation is the preferred approach, with lactic acid bacteria (LAB) commonly added as starter cultures. The International Dairy Federation (IDF), in collaboration with the European Food and Feed Cultures Association, supports this practice and maintains a list of fermentation microorganisms of technological benefit deemed safe for human consumption. The original list included 195 bacterial species and 69 yeast and mould species, while the 2018 update expanded the inventory to 226 bacterial species and 95 fungal species [11].

In a previous study [12], we isolated and identified three LAB strains from spontaneously fermented vegetables, including *Latilactobacillus sakei* F1 and *Lacticaseibacillus rhamnosus* JL from kimchi and *Lacticaseibacillus paracasei* D2 from home-made sauerkraut. These three strains demonstrated increased antioxidant activity in in vitro assays compared to other tested bacterial strains, and the underlying molecular mechanisms responsible for their antioxidant properties were revealed. All belong to bacterial species deemed safe for human consumption on the IDF list [11]. These strains were further assessed by genomic sequencing, which was used to analyse their virulence factors and antibiotic resistance genes [12].

Despite an increasing interest in native Australian foods, the fermentation of indigenous root vegetables has not, to our knowledge, been explored, either through spontaneous fermentations or by use of defined LAB starter cultures. Therefore, the objective of the present study was to develop a novel fermented youlk food product, both by spontaneous fermentation and by using these LAB strains added to the fermentations. Our hypothesis was that use of these strains, each with proven starter activity [12], would enhance the physicochemical properties, improve microbial consistency and stability and increase the final value of the fermented product. This included enhancing the bioactive profile through production of antioxidant compounds, which is known to occur in vitro for these strains [12]. Release of volatile organic compounds (VOCs) and organic acids by the added LAB also results in product improvements; hence, these compounds were also determined as part of this study.

## 2. Materials and Methods

### 2.1. Sample Collection and Ingredients

Youlk tubers were harvested from Dr Geoff Woodall’s youlk cultivation site located in West Arthur, Western Australia. Ground native pepper, used as a spice ingredient in formulations, was sourced from Essential Ingredients, a specialty food supplier based in New South Wales, Australia (Alexandria, NSW, Australia). Non-iodised sea salt (Coles Coarse Sea Salt, Coles Supermarkets Australia Pty Ltd., Melbourne, VIC, Australia) was used for fermentation.

### 2.2. Determination of Nutritional Composition

Fresh youlk was subjected to nutritional analysis by ALS Food and Beverage Pharmaceutical Services (Scoresby, Victoria, Australia). The following methods were used: moisture content was determined using a gravimetric oven-drying method according to AOAC Official Method 934.01 [13]; metal content by inductively coupled plasma optical emission spectrometry (ICP-OES) following nitric acid digestion according to AOAC Official Method 984.27 [13]; total protein by the Kjeldahl method according to AOAC Official Method 992.15 [13]; fat content determined by acid hydrolysis digestion followed by Soxhlet extraction using an internally validated method based on AOAC reference procedures; ash content according to AS 2300.1.5 [14]; and total dietary fibre (TDF) and insoluble dietary fibre (IDF) determined using AOAC Official Methods 985.29 and 991.43, respectively [13]. Soluble dietary fibre (SDF) was calculated by difference (SDF = TDF − IDF), and total carbohydrate by difference following the Food Standards Australia New Zealand (FSANZ) Food Standards Code (Standards 1.2.8 and 1.1.2) method (Carbohydrate (%) = 100 − (% moisture + % protein + % fat + % ash + % TDF).

### 2.3. Preparation of Starter Culture

The bacterial strains used as starters in this study were *Lacticaseibacillus rhamnosus* JL, *Lacticaseibacillus paracasei* D2, and *Latilactobacillus sakei* F1, all isolated from fermented vegetable products [12]. Strains were cultured in Difco^TM^ lactobacilli de Man, Rogosa and Sharpe (MRS) broth (Thermo Fisher Scientific, Scoresby, VIC, Australia). After 24 h of incubation at 37 °C, the LAB cultures reached approximately 10^9^ CFU/mL, as determined by the plate count method. One millilitre of each culture was then centrifuged at 1500× *g* for 10 min at 4 °C using an Eppendorf 5702 R refrigerated centrifuge (Eppendorf AG, Hamburg, Germany). The resulting pellets were washed three times with sterile 0.8% saline to remove residual medium and then resuspended in 1 mL of sterile saline. For fermentation, the bacterial suspension was added at a rate of 1% (*v*/*w*) (equivalent to 1 mL per 100 g of substrate) [15].

### 2.4. Youlk Fermentation

**Figure 1:**
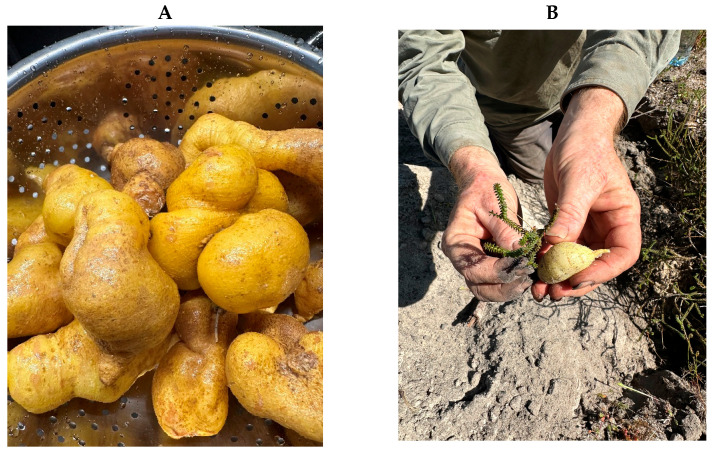
(**A**) rinsed raw youlk (*Platysace deflexa*) and (**B**) freshly harvested youlk.

Youlk tubers were rinsed with tap water to remove surface dirt and contaminants (Figure 1A). Figure 1B shows the freshly harvested youlk. The youlk was then trimmed and cut into 1 cm^3^ cubes. Sterilised anaerobic fermentation jars (100 mL) were used for fermentation. The following ingredients were added to each jar: 50 g youlk cubes, 50 mL sterile water, ground native pepper (1% *w*/*w*), and sea salt at the concentrations shown. For LAB-inoculated fermentation (LAB-inoculated group), eight formulations were prepared with one of three bacterial cultures: JL, D2, F1, and a mixed strain treatment (JL + D2 + F1 in a 1:1:1 ratio), each with 2% or 4% (*w*/*v*) added salt. In addition, two spontaneous fermentations (control group) with 2% or 4% added salt were prepared, using the native microbiota present in the raw materials without the addition of starter cultures. Throughout this study, all treatments (e.g., Mix_2 and Mix_4) were named according to the starter culture composition and salt concentration. All fermentation experiments were conducted in triplicate as independent biological replicates, with each analytical measurement also performed in triplicate. All samples were fermented at room temperature (25 °C) for 48 h to allow active fermentation and then stored in sealed containers at 4 °C for 19 days. Samples were collected aseptically on days 0, 7, 14, and 21 for analyses. For organic acids, supernatants were stored at −80 °C in sterile glass tubes (2 mL; Part No. 5182-0716, Agilent Technologies, Santa Clara, CA, USA) with sealed, airtight lids. For VOC analysis, samples were placed into 20 mL headspace screw vials (Part No. 5188-2753, Agilent Technologies, Santa Clara, CA, USA).

### 2.5. Measurement of pH and Titratable Acidity

The pH of the supernatant in each jar was measured directly using a pH meter (827 pH Lab, Metrohm AG, Heisau, Switzerland). To measure titratable acidity (TA), 2 mL of the supernatant was mixed with 2 mL of distilled water, along with three drops of phenolphthalein indicator. This mixture was then titrated with 0.1 mol/L NaOH (Sigma-Aldrich, St. Louis, MO, USA) until a stable, pale pink colour persisted for 30 s, indicating that the endpoint had been reached, as outlined in AOAC Official Method 942.15. Titratable acidity was calculated as the percentage of lactic acid (% *w*/*v*). All values shown represent the mean ± SD of three replicate samples per treatment group.

### 2.6. Lactic Acid Bacteria Viable Cell Counts and Species Identification Using Matrix-Assisted Laser-Desorption/Ionisation Time-of-Flight Mass Spectrometry (MALDI-TOF MS)

The standard plate count method was used to determine the total number of viable LAB (natural LAB + added starter culture) using lactobacilli MRS agar. A 10 g fermentation sample consisting of both solid and liquid phases (1:1) was aseptically collected, homogenised, and mixed with 40 mL of sterile MRS broth and serially diluted. For each dilution, 100 μL of the sample was plated on MRS agar plates. The plates were incubated at 37 °C for 24–48 h under anaerobic conditions created by AnaeroGen™ sachets (Oxoid) (Thermo Fisher Scientific, Basingstoke, UK, Hampshire, UK). Following incubation, the total CFUs were counted on MRS agar. Bacterial species identification was performed using MALDI-TOF MS with a Bruker MALDI Biotyper system (Bruker Daltonics, Bremen, Germany) following the methods described by Altakhis et al. [16]. Colonies (approximately 10–20 per plate) were selected for identification, ensuring a representative selection of the dominant colony types. Overlapping or closely adjacent colonies were avoided. Data were analysed using the Bruker MALDI Biotyper 4.1.80 software package along with the Bruker library version MBT 8468 MSP Library (Bruker Daltonics GmbH & Co. KG, Bremen, Germany). All experiments were conducted in triplicate.

### 2.7. Determination of Organic Acids by High-Performance Liquid Chromatography (HPLC)

The determination of organic acids, such as lactic acid, citric acid, acetic acid, succinic acid, and malic acid, in samples was conducted using an HPLC system equipped with a diode array detector (DAD) (ACQUITY UPLC^®^, Waters, Milford, MA, USA), according to the method described by Liu et al. [17] with slight modifications. Organic acids were separated using an Aminex HP-87H column (300 × 7.8 mm) from Bio-Rad (Hercules, CA, USA). The mobile phase consisted of 100% 0.003 M sulphuric acid (H_2_SO_4_) (analytical grade, ≥98%, Sigma-Aldrich, St. Louis, MO, USA), with a flow rate of 0.5 mL/min and an oven temperature set to 65 °C. Detection was performed at a wavelength of 210 nm. Brine samples (2 mL) removed from fermentation jars were thawed and centrifuged (Eppendorf AG, Hamburg, Germany) at 6000× *g* for 10 min. The supernatant was passed through a 0.22 µm nylon membrane filter (Millipore, Burlington, MA, USA), and 1 mL of the filtrate was analysed by direct injection. Organic acids were identified and quantified by comparing their retention times and peak areas with those of corresponding HPLC standards. The calibration and identification standards for chromatographic peaks included lactic acid, citric acid, malic acid, succinic acid, and acetic acid, all of which were of analytical grade (≥99%, Merck, Darmstadt, Germany).

### 2.8. Extraction and Detection of VOCs by Headspace Solid-Phase Microextraction (HS-SPME) and Gas Chromatography–Mass Spectrometry (GC-MS)

After fermentation, VOCs were extracted and detected by HS-SPME and GC-MS, respectively. For headspace analysis, 2 mL of the brine sample was homogenised with 2 g of youlk cubes and placed in a 20 mL glass headspace vial (Agilent, Mulgrave, Victoria 3170, Australia). Vials were heated to 40 °C for 30 min with 250 rpm agitation prior to headspace analysis. VOCs were adsorbed onto the SPME fibre (DVB/CAR/PDMS, Agilent Technologies, Part No. 5191-5874; Agilent Technologies, Santa Clara, CA, USA), then the fibre was inserted into a GC-MS (HS-SPME-GC-MS, DSQ9000, Thermo Fisher, USA) fitted with a DB-WAX capillary column (30 m × 250 µm × 0.25 µm; Agilent Technologies, Santa Clara, CA, USA), according to the methods described by Mi et al. [18] with slight modifications.

The GC oven temperature was programmed as follows: initially held at 40 °C for 1 min, then increased to 100 °C at 8 °C/min and held for 3 min, followed by ramp to 150 °C at 5 °C/min and a held for 3 min. The inlet temperature was set to 240 °C, and the gasification chamber was maintained at 250 °C. Helium served as the carrier gas at a flow rate of 1.2 mL/min, with a split ratio of 4:1. Mass spectrometer conditions were as follows: EI ionisation set at 70 eV, ion-source temperature 230 °C, quadrupole temperature 150 °C and a mass scan range of 35–550 *m*/*z*. Volatile compounds were identified by matching the detected mass spectra to the National Institute of Standards and Technology (NIST) Mass Spectral Library (2017 release) using the NIST Mass Spectral Search Program (version 2.4) (National Institute of Standards and Technology, Gaithersburg, MD, USA; https://chemdata.nist.gov/, accessed on 17 January 2026). Retention indices (RIs) were used to support identification. Semi-quantification was performed using the peak area normalisation method, and RIs were calculated according to standard RI definitions [18]. PCA was used to explore the natural clustering patterns of sample data and to visualise differences associated with fermentation time. Partial least squares discriminant analysis (PLS-DA) was applied to determine the variables (VOCs) that most strongly contributed to the separation of samples across fermentation stages. Compounds with a VIP score > 1 in the PLS-DA model were considered key contributors.

### 2.9. Texture Profile Analysis

The texture of samples was evaluated using a TA.XTPlus Texture Analyser (Stable Micro Systems Ltd., Godalming, Surrey, UK). Fermented youlk samples (1 cm^3^) were compressed twice at 50% strain using a flat surface aluminium cylinder probe (35 mm diameter) at a speed of 1.0 mm/s. Texture parameters (hardness, chewiness, cohesiveness, and springiness) were measured three times, and the mean and standard deviations were reported.

### 2.10. Statistical Analysis

Results are expressed as mean ± standard deviation (SD) (*n* = 3). Statistical analyses were performed using Minitab 17 (Minitab, LLC, State College, PA, USA) and GraphPad Prism 10.6.1 (GraphPad Software, Boston, MA, USA) statistical software. A general linear model was used to conduct a two-way analysis of variance (ANOVA), and Tukey’s multiple comparison test (*p* < 0.05) was employed to identify specific differences between treatment means. Principal component analysis (PCA) and partial least squares discriminant analysis (PLS-DA) were performed using R 4.3.0 (R Foundation for Statistical Computing, Vienna, Austria) in RStudio (Posit Software, PBC, Boston, MA, USA). PCA was used to explore the natural clustering patterns of sample data and to visualise differences associated with fermentation time. PLS-DA was applied to determine the variables (VOCs) that most strongly contributed to the separation of samples across fermentation stages and their correlations with metabolite profiles.

## 3. Results

The physicochemical and microbial characteristics of the fermented youlk samples were examined to assess the changes that occur during fermentation. The organic acids, volatile compounds, LAB viability, and texture properties were analysed to characterise the transformations induced by fermentation.

### 3.1. Nutritional Composition of Raw Platysace deflexa

The nutritional analysis of freshly picked youlk (*Platysace deflexa*) in comparison with carrot (*Daucus carota* subsp. *sativus*) and radish (*Raphanus sativus*) is shown in Table 1. Youlk had a higher TDF content (6 g/100 g), of which the majority was IDF (5.0 g/100 g), compared to carrot and radish, while minerals, including sodium, calcium, magnesium, and trace elements, were at lower concentrations.

### 3.2. Changes in the pH Values and Titratable Acidity of Fermented Youlk

**Figure 2:**
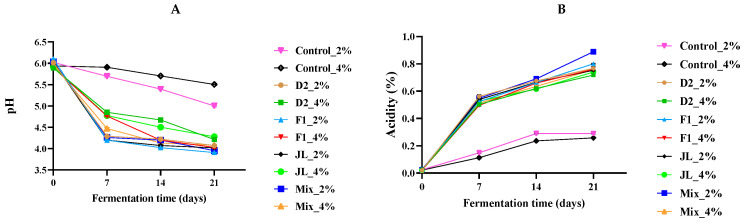
Measurement of the pH (**A**) and acidity (**B**) measured as the TA of each treatment during the spontaneous and LAB-inoculated fermentation period (days 1, 7, 14, and 21).

Changes in the pH values and titratable acidity (TA) of different fermented youlk formulations measured over time are shown in Figure 2A,B. The changes in pH and TA of youlk samples during both LAB-inoculated and spontaneous fermentations were analysed. At the start of fermentation, the pH of all samples ranged from 5.90 to 6.10. Over time, this pH gradually decreased to below 4.5 by the twenty-first day of fermentation. In contrast, the control samples maintained a pH between 5.0 and 5.5 even after 21 days, likely due to reduced microbial activity and a lower rate of acid production. Meanwhile, the mixed culture with 2% salt concentration showed a significantly lower (*p* < 0.05) pH (below 4.00) and higher TA.

The TA of samples increased significantly over time across all treatments (*p* < 0.05). At day 0, all samples exhibited minimal acidity, with a value of less than 0.02%, and no significant differences (*p* < 0.05) were observed among treatments. However, from day 7 onwards, a clear increase in acidity was observed, with the highest values typically recorded at day 21. For instance, the Mix_2% treatment reached the highest acidity level of 0.89 ± 0.00% at day 21, which was significantly higher (*p* < 0.05) than all other treatments. The control group exhibited significantly lower acidity (*p* < 0.05) than the LAB-inoculated group at 7, 14, and 21 days.

### 3.3. Changes in the Concentrations of Organic Acids of Fermented Youlk

**Figure 3:**
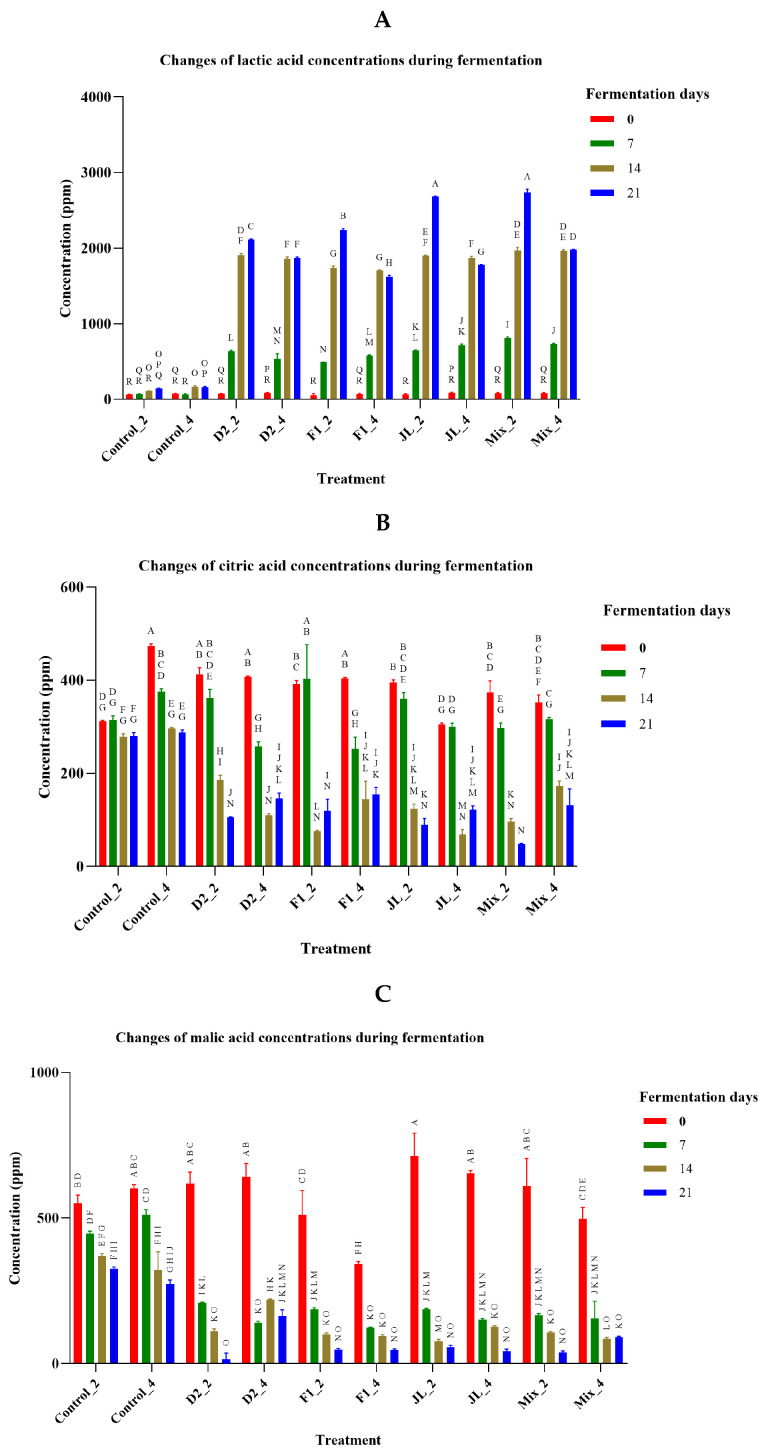

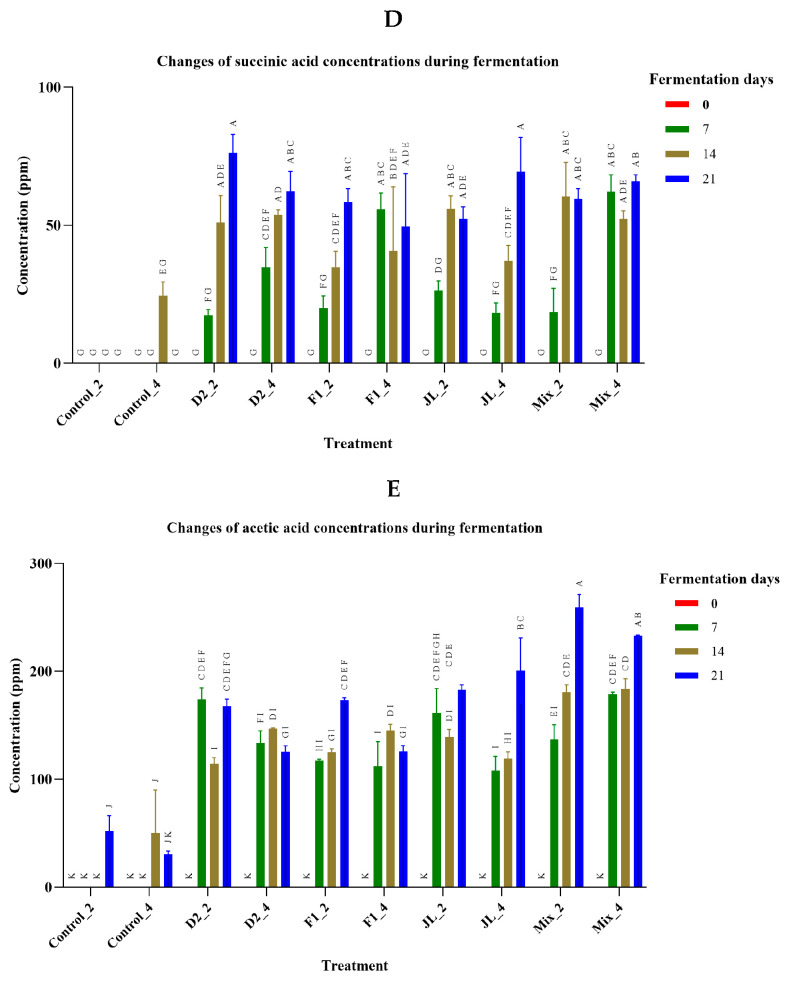
Organic acid concentrations in fermented youlk samples across four fermentation stages (days 0, 7, 14, and 21). Each panel represents an individual organic acid: (**A**) lactic acid, (**B**) citric acid, (**C**) malic acid, (**D**) succinic acid, and (**E**) acetic acid. Values are presented as mean ± SD (*n* = 3). Distinct letters show significant differences among treatments at each time point (*p* < 0.05) according to Tukey’s multiple comparison test.

The organic acid concentrations in the brine samples from different treatments are shown in Figure 3A–E), including lactic acid, succinic acid, malic acid, acetic acid, and citric acid. Across all samples, clear temporal shifts in the major organic acids were observed during fermentation. Citric and malic acids declined continuously from day 0 to day 21 regardless of treatment. At day 0, citric acid levels ranged from 305 to 473.5 ppm, but by day 21, they had decreased to 48–288 ppm, with the lowest levels consistently observed in the LAB-inoculated treatments (especially Mix_2 and D2_2). Malic acid followed a similar decreasing pattern, dropping from initial values of 510–713 ppm to as low as 14–325 ppm by day 21, indicating active microbial utilisation over time except in the control samples. In contrast, lactic acid increased dramatically after fermentation began in the LAB-inoculated group. Day 0 lactic acid values were low (55–89 ppm), and by day 7, lactic acid increased significantly (*p* < 0.05) in all treatments in the LAB-inoculated group, including Mix_2: 810.02 ± 11.6 ppm; Mix_4: 733.37 ± 7.70 ppm; and D2_2: 637.10 ± 11.72 ppm. This rise continued until day 14 and day 21. The control group did not show a considerable increase in lactic acid throughout the fermentation period. Succinic acid, initially absent, emerged from day 7 onwards and increased gradually, particularly in the D2_2, D2_4, JL_2, and Mix treatments. By day 21, succinic acid levels ranged from 52 to 76 ppm in most LAB-inoculated samples. Similarly, acetic acid was not detected at day 0 but appeared on day 7, with the highest levels consistently found in the Mix and JL treatments. On day 21, acetic acid peaked in Mix_2 (259.24 ± 11.96 ppm) and Mix_4 (232.89 ± 9.2 ppm), while controls produced only minor amounts (30.50 ± 2.91–52.16 ± 13.98 ppm).

### 3.4. Microbiological Analyses

**Figure 4:**
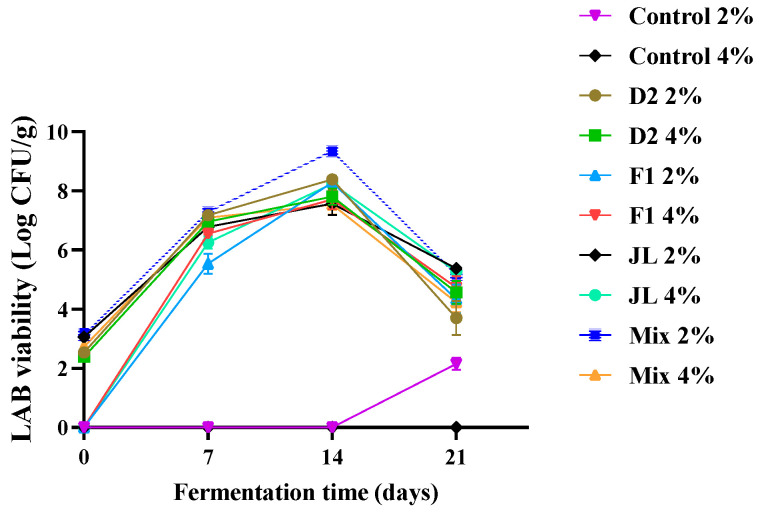
The variations in viable LAB (log CFU/g) during youlk fermentation (days 1, 7, 14, and 21).

The presence and growth progression of lactic acid bacteria in the ten treatments showed distinct trends during fermentation, as shown in Figure 4. At day 0, LAB were undetectable in the control 2%, F1_2%, F1_4%, and JL_4% formulations. However, other LAB-inoculated treatments initially showed low populations of 2.39 to 3.14 log CFU/g. Interestingly, at all time points (days 0, 7, 14, and 21), *Pluralibacter gergoviae*, an opportunistic Gram-negative microorganism belonging to the Enterobacteriaceae family, was detected in the control group. Additionally, *Weissella cibaria* (Leuconostocaceae) was detected in the Control_2% treatment (2.15 ± 0.21 log CFU/g) at day 21. Meanwhile, the LAB-inoculated group showed *Weissella cibaria* on days 7, 14, and 21 alongside the inoculated LAB strains, while *P. gergoviae* was notably inhibited. The F1 strain did not exhibit any growth until day 7. Overall, the highest growth of LAB was observed on days 7 and 14 across all samples in the LAB-inoculated group.

### 3.5. Changes in the Texture of Fermented Youlk

The textural properties of the samples, including hardness, cohesiveness, springiness, and chewiness, were influenced by both the formulation and the fermentation time, as shown in Table S2 (Supplementary Materials). Generally, hardness significantly changed over time across all treatments. On day 0, hardness values (gf) ranged from 18,227.17 ± 2174.08 to 24,763.22 ± 547.96, whereas on day 21, the values decreased to 10,302.99 ± 369.62 to 14,962.69 ± 1708.67. The highest hardness values were observed on day 0, with progressively lower values at later timepoints. Cohesiveness and springiness remained relatively stable, with no significant differences (*p* < 0.05) across time points in most treatments. Overall, chewiness decreased in parallel with hardness. On day 0, chewiness (gf) ranged from 2434.25 ± 911.81 to 5788.63 ± 584.18, while on day 21, it ranged from 1061.51 ± 1462.53 to 2078.83 ± 819.38.

### 3.6. Analysis of Volatile Organic Compounds (VOC)

**Figure 5:**
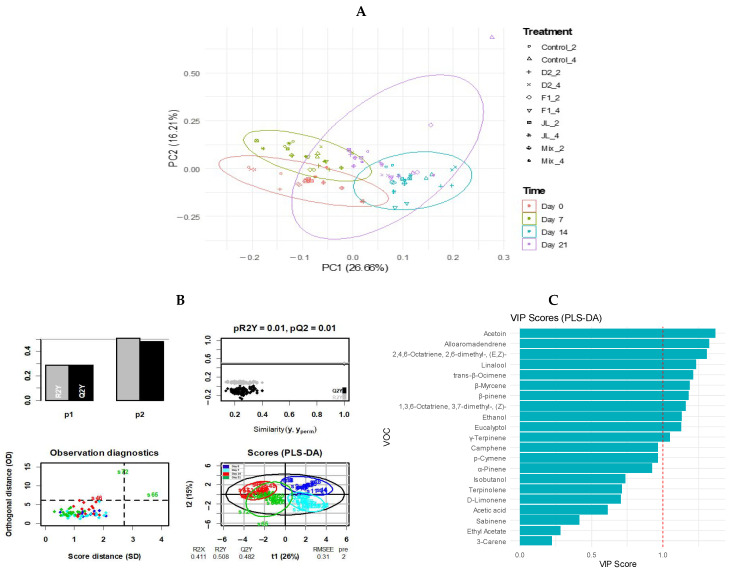
(**A**) Principal component analysis (PCA) score plot derived from GC-MS data of fermented youlk samples for 21 days. (**B**) Summary of the PLS-DA model, including model performance, validation, diagnostics, and sample separation. (**C**) The variable importance in projection (VIP) value of volatile substances.

VOCs detected by SPME-GC/MS analysis of fermented youlk samples with different formulations after 0, 7, 14, and 21 days are shown in Table S1, together with PCA and PLS(DA) analyses of these data in Figure 5. A total of 21 VOCs were identified during the fermentation of youlk. As shown in Figure 5A, PCA was performed to visualise differences in the volatile profiles across fermentation time. Overall, the PCA revealed a clear time-dependent pattern from day 0 to day 21, confirming that fermentation time significantly influenced the volatile composition and led to progressive differentiation of the samples.

The partial least squares discriminant analysis (PLS-DA) model was developed as shown in Figure 5B to discriminate among the time points based on the 21 predictor variables (% of relative abundance of 21 VOCs). The model exhibits satisfactory performance, with a cumulative R^2^Y value of 0.508. As shown in Figure 5C, the VIP score of each compound, acetoin, alloaromadendrene, ‘2,4,6-octatriene, 2,6-dimethyl-, (E,Z)-’, linalool, trans-β-ocimene, β-myrcene, β-pinene, ‘1,3,6-octatriene, 3,7-dimethyl-, (Z)-’, ethanol, eucalyptol and γ-terpinene, is higher than 1. Therefore, these VOCs have a greater impact on distinguishing the fermentation stage based on their relative abundance.

As shown in Table S1, compounds with VIP > 1 were extracted from the PLS-DA model and subjected to ANOVA to evaluate treatment effects. Despite their importance in the multivariate model, ANOVA revealed that most VOCs did not show substantial or statistically significant differences across treatments. However, consistent with the PLS-DA results, the VOC profiles changed markedly over the fermentation period, and most treatments exhibited a similar temporal pattern. Fermentation time strongly influenced the VOC profiles. Acetoin exhibited a distinct early peak, with the highest levels observed at day 14, followed by a decline at day 21 (*p* < 0.05). Alloaromadendrene decreased steadily across all time points. In contrast, 2,4,6-octatriene (2,6-dimethyl-, (E,Z)-) exhibited a clear mid-fermentation maximum, being nearly absent at days 0 and 7, reaching its highest value at day 14, and decreasing again by day 21 (*p* < 0.05) (Table S1). trans-β-Ocimene increased later in fermentation, with significantly higher levels at days 14 and 21 compared with days 0 and 7 (*p* < 0.05). β-Myrcene, β-pinene and 1,3,6-octatriene, 3,7-dimethyl-, (Z)- all peaked at day 7 and declined thereafter (*p* < 0.05). Eucalyptol also reached its maximum at initial stage of fermentation. Overall, fermentation time had a stronger impact than treatment effects, which remained minimal for most compounds.

Several plant-derived monoterpenes also exhibited treatment-related variation, in addition to their time-dependent changes. Linalool levels were highest at day 14 and declined slightly by day 21; Mix_2 generally retained higher levels, while Control_2 showed the lowest. Fermentation metabolites followed expected patterns as well: ethanol was nearly absent at day 0, increased markedly at days 7 and 14, and reached its highest mean level at day 21 (*p* < 0.05), with Mix_2 producing the highest overall ethanol concentration among treatments.

## 4. Discussion

Aboriginal populations in Australia have historically maintained health through culturally grounded dietary practices [20,21]. These diets are diverse, minimally processed and sustainable, being closely aligned with both the natural environment and cultural traditions, and include a wide range of seeds, nuts, over 150 varieties of tubers and roots, and more than 300 types of fruits and vegetables [22,23,24]. Notwithstanding this, only 25 species of approximately 6500 native food plants have been approved for commercial sale by Food Standards Australia New Zealand (FSANZ) [25]. The aim of this study was to determine if fermentation by lactic acid bacteria (LAB) could improve the properties of a native vegetable, youlk (*Platysace deflexa*), to enhance its dietary and commercial potential.

In many traditional fermented vegetable products, fermentation primarily occurs through the action of naturally occurring LAB present in raw ingredients, although commercial producers generally prefer to use added LAB and/or other cultures. Their growth and activity are influenced by several factors, including ingredient composition, fermentation temperature, and salt concentration. This study investigated the production of a novel fermented vegetable product from a native Australian vegetable, youlk. Additionally, it compared the fermentation behaviour of naturally fermented (control) youlk with that of youlk inoculated with three selected LAB strains either alone or in combination. The use of LAB strains with proven antioxidant activity could enhance the antioxidant properties of fermented vegetables [26,27]. However, the antioxidant activity of the fermented youlk products in this study has not yet been experimentally confirmed and will be addressed in future work. The fermentation process was carried out at room temperature (25 °C) for 48 h to support active fermentation, followed by storage at 4 °C for 19 days to simulate refrigerated storage and slow fermentation. LAB activity was not expected to be inhibited completely during refrigeration; rather, limited metabolic activity may have continued, as previous fermented vegetable studies have shown that while lower temperature delays acidification and ripening, viable LAB can persist during refrigerated storage [28,29,30].

The aim of including ground native pepper (1% *w*/*w*) in the formulation was to improve the product’s sensory appeal and functional characteristics given its recognised use as a native spice and source of bioactive compounds, including those with antimicrobial properties [31]. Its application at low concentration was not expected to inhibit fermentation, though may have caused some selective pressure on microbial communities. Preliminary optimisation trials were conducted at concentrations ranging from 0.5% to 3% *w*/*w* to determine a suitable concentration range, and 1% was chosen as an appropriate level that allowed fermentation to proceed while maintaining desirable product characteristics.

Regarding the nutritional analysis of youlk, it is evident that youlk contains a higher amount of IDF than well-known tuber vegetables, such as carrot and radish. IDF, in particular, supports healthy gastrointestinal activity by stimulating intestinal movement (peristalsis) and increasing stool bulk, which helps maintain regular bowel function [32].

pH and TA are key indicators in vegetable fermentation, as they influence both microbial growth and the flavour of fermented products through the accumulation of fermentation metabolites [33]. In the present study, only youlk inoculated with LAB strains exhibited a rapid pH drop by the end of fermentation (3.95 ± 0.01 for Mix_2, 4.62 ± 0.02 for D2_4), indicating that microbial growth, especially LAB growth, occurred, while in the control samples, only very limited (or no) fermentation occurred. A reduction in pH accompanied the consistent increase in titratable acidity, demonstrating the accumulation of organic acids throughout fermentation [34]. By the end of the fermentation process, TA was significantly (*p* < 0.05) higher in culture-dependent samples, which ranged from 0.72 ± 0.01% to 0.89 ± 0.00% lactic acid equivalent, than in the control, which showed 0.29 ± 0.00 and 0.26 ± 0.00 in Control_2 and Control_4 samples, respectively, confirming that LAB was mostly activated in culture-dependent samples.

The metabolism of sugar substrates by both heterofermentative and homofermentative lactic acid bacteria, such as *Lactobacillus*, *Leuconostoc*, *Lactococcus*, *Weissella*, and *Pediococcus*, has been consistently observed in various vegetable fermentation processes, and this metabolism leads to the production of lactic acid and acetic acid [35,36]. In our study, initial lactic acid levels were low, ranging from 55 to 89 ppm on day 0. However, by day 7, lactic acid concentrations rose significantly across the LAB-inoculated group. Organic acids, particularly lactic and acetic acid, are major metabolites produced by LAB and function as important quality markers in kimchi, influencing both its characteristic flavour and microbial safety [37]. In this study, it was found that, aside from lactic acid, the concentrations of malic and citric acids generally decreased during fermentation. Lactic acid production is associated with malic acid depletion where malolactic fermentation (MLF) occurs in fermentations [38,39,40,41], although MLF cannot be conclusively confirmed in the youlk fermentations based solely on these results, as lactic acid production may also result from carbohydrate conversion [42]. Notwithstanding this, it is intriguing that *Weissella* populations (which perform MLF) increased in the inoculated samples. Wu et al. [43] revealed that lactic acid was the predominant organic acid detected in pickled wax gourd, whereas acetic acid remained at relatively low concentrations. Similar to those findings, the current study found that the LAB-inoculated youlk exhibited higher levels of lactic acid and relatively low levels of acetic acid, confirming a strong correlation between titratable acidity and lactic acid content. As the predominant organic acid formed during fermentation, lactic acid may intensify the fermented food flavour, enhance microbial stability, and contribute to greater consumer acceptability. Unlike fermented youlk that used the added LAB cultures, the spontaneously fermented control group did not show an increase in lactic acid. This indicates that adding starter-type LAB cultures to youlk enhances the spontaneous fermentation in a beneficial way.

The species identified by MALDI-TOF MS correspond to the dominant colonies selected from the total viable counts obtained using MRS agar, thereby reflecting the dominant culturable LAB present in the samples. It is important to note that these identification results are qualitative and do not directly correspond to the total LAB counts shown in Figure 4. Viable counts revealed a progressive increase in LAB populations in the LAB-inoculated youlk samples. Generally, vegetable fermentations are characterised by a rapid increase in LAB, a marked accumulation of lactic acid and a pronounced decline in pH. In contrast, Enterobacteriaceae typically dominate only at the early stages and are rapidly outcompeted once LAB-driven acidification proceeds [44]. LAB are essential to vegetable fermentation, such as kimchi, because they produce lactic and acetic acids that effectively inhibit the growth of spoilage organisms and pathogenic bacteria [45]. The use of LAB strains with antioxidant activity in this study may contribute to antioxidant benefits in the final product; however, this potential benefit requires further investigation. Surprisingly, some inoculated treatments (F1_2%, F1_4% and JL_4%) and one control (2%) showed no detectable LAB on day 0. This could be due in part to the presence of salt and matrix conditions including native pepper that may have stressed the inoculated LAB, affecting their recovery and/or [46,47]. In addition, in the control group, lactic acid fermentation did not become established, as evidenced by the absence of detectable LAB throughout fermentation and only a very low *W. cibaria* population (2.15 ± 0.21 log CFU/g), observed in Control_2 at day 21, which likely represents residual survival rather than active fermentation. In addition, *P. gergoviae* was detected in the control group on days 0, 7, and 14, and 21. In the LAB-inoculated group, we observed that *P. gergoviae* growth was inhibited. Furthermore, the findings of this study indicated that the *W. cibaria* species were present in nearly all treatment groups on days 7, 14, and 21, suggesting that they may be part of the natural microbiota found in youlk. In addition to the added LAB, only *P. gergoviae* and *W. cibaria* were detected in the samples; however, this may be attributed to the use of MRS agar, a selective medium that would have limited the detection of other bacterial species present. Our results revealed that the Mix_2 treatment resulted in a desirable fermentation, in line with the study conducted by Park et al. [48], who found that multi-strain LAB starter cultures can accelerate fermentation and improve product quality. The greater LAB numbers in the inoculated samples in our study presumably led to enhanced metabolic activity, thereby increasing the production of organic acids with a decline in pH and an increase in titratable acidity. Furthermore, the observation of low levels of *W. cibaria* at day 21, along with the organic acid profile, pH, and titratable acidity of the Control_2 sample, which remained similar to their initial values, suggests that the fermentation process was less effective compared to the groups with the added LAB. The microbial community composition in this study was not determined using DNA-based methods such as 16S rRNA gene sequencing, although this would have yielded a more comprehensive picture of the species composition. Further work is being planned along these lines, together with sensory evaluation of the fermented product.

A gradual decrease in hardness and chewiness was observed in the LAB-inoculated group and the control group as shown in Table S2. This is likely due to natural texture-softening mechanisms such as moisture loss, endogenous pectin-degrading enzyme activity, and storage-related tissue senescence. Even in the absence of LAB fermentation, these physicochemical changes may progressively weaken the cell wall structure, leading to reduced firmness over time, while both springiness and cohesiveness remained unchanged during fermentation. Yang et al. [33] found that radish paocai softened markedly during fermentation, with firmness and chewiness decreasing over a seven-day period, while cohesiveness remained relatively unchanged. However, the same study found that the decline in chewiness contrasts with the study conducted by Rao et al. [49], who reported an increase in chewiness during radish fermentation, indicating that differences may reflect variations in radish type and microbial communities. In addition, Ao et al. [50] identified pH, salt levels, and enzymatic activity as major factors influencing radish paocai texture. Meanwhile, Hernández et al. [51] demonstrated that fungal metabolism can further degrade pectin and sugars, thereby contributing to texture softening.

The characteristic aroma of fermented vegetables is primarily influenced by a diverse array of volatile compounds, including esters, aldehydes, ketones, sulphur-containing compounds, phenols, and alcohols [52]. The key VOCs generated during youlk fermentation are essential for developing a product with appealing flavours. This study explored the VOCs present in fermented youlk, comparing samples with and without starter cultures. The findings revealed a clear correlation between volatile compounds and the progression of fermentation over time regardless of whether a starter culture was used. This suggests that although LAB are involved in the production of VOCs, the contribution of this specific strain to the overall volatile profile is minimal. Similar results were reported in the study by Kraouia et al. [52] on sea-fennel-incorporated kimchi, which found that treatment-based separation was minimal. VOCs are primarily formed through chemical oxidation and enzymatic reactions during fermentation. Overall, this study revealed that the fermented youlk contained a wide range of terpenes, such as α-pinene, β-pinene, sabinene, 3-carene, β-myrcene, D-limonene, eucalyptol, trans-β-Ocimene, γ-terpinene, camphene, 1,3,6-Octatriene, 3,7-dimethyl-, (Z)-, terpinolene, 2,4,6-Octatriene, 2,6-dimethyl-, (E,Z)-, and alloaromadendrene. The majority of them may have arisen due to the presence of native pepper [53,54]. Sulphides are regarded as important aroma-active compounds in fermented foods, and variations in their levels can strongly influence consumer acceptance or rejection [17,55]. However, in this study, sulphur-containing compounds were not detected. This absence may be attributed primarily to the metabolic activity of the microbial community, although analytical and precursor-related factors cannot be excluded. Area normalisation was applied to express VOC abundance as relative percentage values, a widely accepted approach for semi-quantitative VOC analysis that avoids the need for compound-specific calibration curves. Therefore, PCA and PLS-DA were performed only across fermentation time points, with VIP-selected (VIP > 1) VOCs subsequently subjected to univariate ANOVA by treatment to assess culture-specific effects on the key discriminatory compounds. Overall, the results showed that most terpenes remained stable throughout fermentation, while acetoin levels increased over time, a desirable feature in fermented foods [42]. Wang et al. [42] mentioned that in LAB-fermented fruit and vegetable juices, the concentrations of acetoin can reach high levels. This demonstrates that LAB fermentation can achieve high acetoin yields even with plant-based substrates. However, the inherent complexity of plant matrices, combined with multiple interacting metabolic pathways and fermentation-dependent factors such as temperature, pH, and aeration, can influence VOC dynamics. In addition, ethanol was the most abundant alcohol in the fermented youlk, which may enhance the flavour of the final product. In our study, the ethanol content in the Mix_2 treatment increased significantly, rising from 0.09 ± 0.00% on day 0 to 2.95 ± 0.27% by day 21, marking the highest increase among all treatments. A similar observation was reported by Hong et al. [56], who found that ethanol was increased, which accounted for more than 93% of alcoholic compounds during kimchi fermentation, which was conducted at 4 °C in 35 days, while Lee et al. [57] observed a decline in alcohol during kimchi fermentation at 15 °C. Kraouia et al. [52] suggested that despite variations in starter cultures, the ultimate volatile characteristics of kimchi are predominantly shaped by environmental parameters, particularly temperature and ingredient composition. In this study, α-pinene was the most abundant VOC. This compound has reported antimicrobial, anticancer, anti-inflammatory, and antiallergic properties [58], which may provide additional advantage for the product. During fermentation, the levels of acetoin, ethanol, and linalool increased compared to other volatile compounds. Ethanol was the predominant alcohol detected in optimally fermented kimchi [56] and has been reported to show only a limited correlation with overall sensory preference [59]. Linalool imparts fruity and floral notes, contributing positively to the aroma profile of fermented foods [41]. Furthermore, the increase in acetoin during fermentation is considered desirable, as it enhances flavour through its characteristic buttery notes [42].

## 5. Conclusions

Youlk exhibited high levels of IDF, which may support gut health and positively influence fermentation performance. LAB-inoculated fermentation successfully established lactic acid fermentation, with added LAB leading to more rapid pH reduction, increased LAB counts and effective inhibition of *P. gergoviae*. Fermentation time had a greater impact on metabolite variation than the choice of starter culture, with lactic acid, α-pinene, ethanol, linalool, and acetoin identified as the predominant compounds among organic acids and VOCs. These results indicate that youlk exhibits favourable fermentation characteristics. Overall, LAB-inoculated fermentation provides advantages over spontaneous fermentation. Future work should include sensory validation of the fermented products and investigation of the synergistic effects of spices and herbs to further enhance flavour and nutritional value.

## Figures and Tables

**Table 1:** Nutritional analysis of youlk (*Platysace deflexa*), compared with carrot (*Daucus carota* subsp. *sativus*) and radish (*Raphanus sativus*) per 100 g (fresh weight). Nutritional components of youlk were determined in this study (see Materials and Methods), whereas the values for carrot and radish were obtained from the Australian Food Composition Database (FSANZ, 2019) [19].

Component	Youlk	Carrot	Radish
Moisture (g)	89.1	89	92.9
Ash (g)	0.6	0.9	0.6
Carbohydrates (g)	4.6	6.6	2.9
Total Fat (g)	0.2	0	0.3
Protein (g)	0.5	0.6	0.7
TDF (g)	6	3.2	1.8
SDF (g)	<1	NS	NS
IDF (g)	5.0	NS	NS
Minerals (mg)			
Potassium	140	266	210
Sodium	56	51	28
Calcium	46	26	30
Magnesium	28	11	13
Phosphorus	14	31	23
Manganese	<1	0.148	NS
Zinc	<1	0.15	0.5

NS, not stated. TDF: total dietary fibre; SDF: soluble dietary fibre; IDF: insoluble dietary fibre.

## Data Availability

The original contributions presented in this study are included in the article/Supplementary Materials. Further inquiries can be directed to the corresponding author.

## Supplementary Materials

The following supporting information can be downloaded at: https://www.mdpi.com/article/10.3390/foods15111973/s1, Table S1: VOCs (relative area normalisation of chromatographic peak, mean% ± SD) with VIP > 1 selected from the PLS-DA model and their ANOVA results across different treatments; Table S2: Changes in texture of fermented youlk (*n* = 3).

## Abbreviations

The following abbreviations are used in this manuscript:

LAB	Lactic Acid Bacteria
VOCs	Volatile Organic Compounds
RIRDC	Rural Industries Research and Development Corporation
IDF	International Dairy Federation
ICP-OES	Inductively Coupled Plasma Optical Emission Spectrometry
TA	Titratable Acidity
HPLC	High-Performance Liquid Chromatography
MALDI-TOF MS	Matrix-Assisted Laser-Desorption/Ionisation Time-Of-Flight Mass Spectrometry
HS-SPME	Headspace Solid-Phase Microextraction
TDF	Total Dietary Fibre
IDF	Insoluble Dietary Fibre
SDF	Soluble Dietary Fibre
FSANZ	Food Standards Australia New Zealand
MLF	Malolactic Fermentation
