# Supplementary material for: Fermentation of Youlk, an Australian Native Root Vegetable, Using Defined Lactic Acid Bacterial Strains
Source: Foods. 2026 Jun 2;15(11):1973. doi: 10.3390/foods15111973 (PMC13257415; doi:10.3390/foods15111973)
Supplement: Supplementary file 1 [file foods-15-01973-s001.zip › foods-4271683-supplementary.pdf]

Supplementary Table S1: VOCs (relative area of chromatographic peak, mean%±SD) with VIP > 1 selected from the PLS-DA model and their ANOVA results across different treatments.

Day	Treatment	Acetoin	Alloaromadendrene	2,4,6-Octatriene, 2,6-dimethyl-, (E,Z)-	Linalool	trans-β-Ocimene	β-Myrcene	β-pinene	1,3,6-Octatriene, 3,7-dimethyl-, (Z)-	Ethanol	Eucalyptol	γ-Terpinene
0	Control_2	0.09±0.00^{c}	0.08±0.00^{ab}	0.00±0.00^{f}	0.07±0.00^{i-l}	0.08±0.00^{f-n}	3.27±0.28^{a-d}	2.86±0.15^{abc}	1.79±0.09^{a}	0.10±0.00^{b}	0.75±0.04^{abc}	0.06±0.00^{abc}
	Control_4	0.10±0.00^{c}	0.06±0.00^{b-f}	0.00±0.00^{f}	0.07±0.01^{i-l}	0.03±0.00^{n}	1.94±0.02^{b-i}	2.51±0.01^{a-g}	0.70±0.00^{d-j}	0.10±0.00^{b}	0.49±0.00^{bc}	0.10±0.00^{abc}
	D2_2	0.02±0.00^{c}	0.08±0.00^{ab}	0.00±0.00^{f}	0.05±0.00^{jkl}	0.04±0.00^{mn}	2.43±0.00^{a-g}	3.03±0.01^{ab}	1.02±0.01^{b-e}	0.08±0.00^{b}	0.38±0.00^{bc}	0.05±0.00^{bc}
	D2_4	0.00±0.00^{c}	0.08±0.00^{ab}	0.00±0.00^{f}	0.09±0.01^{f-l}	0.08±0.00^{f-n}	3.60±0.04^{a}	3.17±0.04^{a}	1.50±0.00^{ab}	0.06±0.00^{b}	0.44±0.01^{bc}	0.06±0.00^{abc}
	F1_2	0.00±0.00^{c}	0.05±0.00^{d-i}	0.00±0.00^{f}	0.05±0.00^{jkl}	0.04±0.00^{mn}	2.41±0.11^{a-h}	2.69±0.01^{a-e}	0.57±0.01^{d-j}	0.08±0.00^{b}	0.50±0.01^{bc}	0.07±0.01abc
	F1_4	0.02±0.00^{c}	0.04±0.00^{f-l}	0.00±0.00^{f}	0.05±0.01^{jkl}	0.03±0.00^{n}	2.23±0.14^{a-i}	2.62±0.05^{a-e}	0.49±0.02^{e-j}	0.06±0.00^{b}	0.43±0.01^{bc}	0.04±0.00^{bc}
	JL_2	0.02±0.00^{c}	0.04±0.00^{e-k}	0.00±0.00^{f}	0.07±0.01^{h-l}	0.04±0.00^{n}	2.35±0.03^{a-i}	2.74±0.07^{a-e}	0.51±0.00^{d-j}	0.06±0.00^{b}	0.44±0.00^{bc}	0.05±0.00^{bc}
	JL_4	0.00±0.00^{c}	0.05±0.00^{c-h}	0.00±0.00^{f}	0.09±0.01^{f-l}	0.06±0.00^{k-n}	1.56±0.05^{e-i}	1.32±0.03^{g-k}	0.94±0.06^{c-f}	0.01±0.00^{b}	0.34±0.01^{c}	0.03±0.00^{bc}
	Mix_2	0.04±0.00^{c}	0.06±0.00^{b-g}	0.00±0.00^{f}	0.09±0.01^{e-l}	0.04±0.00^{mn}	2.06±0.05^{a-i}	2.12±0.03^{a-h}	0.94±0.10^{c-f}	0.09±0.00^{b}	0.83±0.08^{abc}	0.04±0.00^{bc}
	Mix_4	0.04±0.00^{c}	0.04±0.00^{f-m}	0.00±0.00^{f}	0.08±0.00^{g-l}	0.04±0.00^{n}	2.50±0.08^{a-g}	2.68±0.02^{a-e}	0.47±0.02^{e-j}	0.08±0.00^{b}	0.80±0.09^{abc}	0.04±0.00^{bc}
7	Control_2	0.04±0.01^{c}	0.03±0.00^{i-n}	0.00±0.00^{f}	0.03±0.00^{l}	0.07±0.01^{i-n}	2.95±0.73^{a-e}	2.98±0.06^{ab}	1.50±0.05^{ab}	0.07±0.03^{b}	1.05±0.02^{abc}	0.14±0.01^{abc}
	Control_4	0.09±0.04^{c}	0.03±0.00^{g-n}	0.00±0.00^{f}	0.05±0.03^{jkl}	0.06±0.00^{k-n}	2.96±0.08^{a-e}	2.51±0.06^{a-g}	1.01±0.08^{b-e}	0.09±0.00^{b}	1.68±0.05^{ab}	0.08±0.00^{abc}
	D2_2	0.81±0.05^{bc}	0.04±0.00^{e-j}	0.00±0.00^{f}	0.04±0.00^{kl}	0.05±0.00^{lmn}	2.70±0.03^{a-g}	2.31±0.03^{a-h}	0.87±0.06^{c-g}	1.60±0.00^{ab}	1.02±0.03^{abc}	0.06±0.01^{abc}
	D2_4	0.19±0.00^{bc}	0.04±0.01^{f-l}	0.00±0.00^{f}	0.03±0.00^{l}	0.05±0.00^{k-n}	2.30±0.34^{a-i}	1.83±0.29^{b-k}	1.05±0.10^{bcd}	1.88±0.03^{ab}	1.96±0.24^{a}	0.08±0.01^{abc}
	F1_2	0.07±0.06^{c}	0.04±0.00^{f-m}	0.00±0.00^{f}	0.11±0.07^{e-l}	0.06±0.00^{k-n}	2.26±0.29^{a-i}	2.82±0.12^{a-d}	1.35±0.07^{abc}	1.76±0.00^{ab}	0.87±0.04^{abc}	0.07±0.00^{abc}
	F1_4	0.42±0.07^{bc}	0.02±0.00^{j-n}	0.00±0.00^{f}	0.07±0.00^{h-l}	0.06±0.01^{j-n}	2.46±0.21^{a-g}	2.54±0.15^{a-f}	0.94±0.06^{c-f}	1.67±0.03^{ab}	1.26±0.04^{abc}	0.10±0.01^{abc}
	JL_2	0.34±0.14^{bc}	0.05±0.01^{d-h}	0.00±0.00^{f}	0.03±0.00^{l}	0.08±0.01^{h-n}	3.49±0.04^{ab}	2.84±0.02^{a-d}	1.82±0.17^{a}	1.64±0.01^{ab}	1.03±0.05^{abc}	0.16±0.02^{ab}
	JL_4	0.97±0.08^{abc}	0.04±0.00^{f-m}	0.00±0.00^{f}	0.05±0.01^{jkl}	0.08±0.01^{h-n}	3.35±0.20^{abc}	2.89±0.22^{abc}	1.55±0.21^{ab}	1.48±0.06^{ab}	1.32±0.05^{abc}	0.12±0.02^{abc}
	Mix_2	1.07±0.13^{abc}	0.03±0.00^{f-m}	0.00±0.00^{f}	0.12±0.00^{d-k}	0.08±0.00^{g-n}	2.54±0.95^{a-g}	2.79±0.05^{a-e}	1.51±0.10^{ab}	1.82±0.25^{ab}	0.95±0.07^{abc}	0.08±0.01^{abc}
	Mix_4	0.19±0.01^{bc}	0.03±0.00^{h-n}	0.00±0.00^{f}	0.10±0.02^{e-l}	0.06±0.00^{k-n}	2.78±0.10^{a-f}	2.75±0.14^{a-e}	0.82±0.05^{c-h}	1.57±0.00^{ab}	1.32±0.10^{abc}	0.07±0.01^{abc}
14	Control_2	0.05±0.00^{c}	0.03±0.00^{i-n}	0.08±0.09^{ab}	0.09±0.01^{e-l}	0.14±0.01^{c-j}	1.85±0.25^{c-i}	1.76±0.05^{c-k}	0.58±0.09^{d-j}	0.09±0.04^{b}	0.83±0.08^{abc}	0.07±0.00^{abc}
	Control_4	0.08±0.00^{c}	0.02±0.00^{j-n}	0.02±0.02^{cd}	0.09±0.00^{a-d}	0.13±0.01^{c-k}	1.32±0.42^{f-i}	0.81±0.07^{jk}	0.42±0.02^{f-j}	0.07±0.21^{b}	1.52±0.34^{abc}	0.02±0.02^{bc}
	D2_2	0.14±0.00^{bc}	0.02±0.00^{lmn}	0.40±0.02^{cd}	0.16±0.00^{b-g}	0.11±0.01^{e-n}	1.35±0.08^{f-i}	1.28±0.30^{h-k}	0.40±0.02^{f-j}	2.61±0.00^{abc}	0.76±0.02^{abc}	0.03±0.01^{bc}
	D2_4	0.42±0.04^{bc}	0.02±0.00^{j-n}	0.49±0.01^{bc}	0.13±0.00^{d-j}	0.13±0.00^{c-k}	1.63±0.01^{e-i}	0.78±0.37^{k}	0.49±0.01^{e-j}	2.65±0.00^{abc}	1.13±0.09^{abc}	0.05±0.00^{bc}
	F1_2	0.39±0.26^{bc}	0.07±0.01^{a-e}	0.32±0.03^{de}	0.21±0.00^{a-d}	0.15±0.01^{b-h}	0.85±0.12^{hi}	1.41±0.01^{f-k}	0.32±0.03^{hij}	2.85±0.08^{abc}	0.46±0.13^{bc}	0.00±0.00^{c}

	F1_4	0.57 ± 0.22^{bc}	$0.07\pm0.00^{a-d}$	0.21 ± 0.00^{e}	$0.17\pm0.01^{b-f}$	0.35 ± 0.03^{a}	$1.45\pm0.20^{e-i}$	$1.66\pm0.08^{d-k}$	0.21 ± 0.00^{j}	2.68 ± 0.15^{abc}	0.48 ± 0.20^{bc}	0.00 ± 0.00^{c}
	JL_2	1.00 ± 0.06^{abc}	0.02 ± 0.00^{lmn}	0.35 ± 0.05^{d}	$0.18\pm0.00^{b-e}$	$0.11\pm0.01^{e-m}$	$1.42\pm0.13^{e-i}$	$1.71\pm0.11^{c-k}$	$0.35\pm0.05^{g-j}$	2.53 ± 0.03^{abc}	0.67 ± 0.04^{abc}	0.05 ± 0.00^{bc}
	JL_4	1.00 ± 0.06^{abc}	$0.02\pm0.00^{j-n}$	0.58 ± 0.08^{ab}	0.25 ± 0.01^{ab}	$0.15\pm0.02^{b-g}$	$1.46\pm0.24^{e-i}$	$1.60\pm0.49^{e-k}$	$0.58\pm0.08^{d-j}$	2.68 ± 0.07^{abc}	0.53 ± 0.27^{bc}	0.03 ± 0.01^{bc}
	Mix_2	2.07 ± 0.49^{a}	$0.02\pm0.00^{j-n}$	0.65 ± 0.00^{a}	0.22 ± 0.00^{abc}	$0.16\pm0.00^{b-e}$	$1.69\pm0.00^{d-i}$	$1.89\pm0.00^{b-k}$	$0.65\pm0.00^{d-j}$	2.91 ± 0.08^{abc}	0.80 ± 0.00^{abc}	0.04 ± 0.00^{bc}
	Mix_4	0.95 ± 0.07^{abc}	$0.02\pm0.00^{j-n}$	0.61 ± 0.02^{ab}	$0.15\pm0.00^{c-i}$	$0.14\pm0.01^{b-i}$	$1.73\pm0.03^{d-i}$	$1.91\pm0.14^{b-k}$	$0.61\pm0.02^{d-j}$	2.60 ± 0.12^{abc}	1.16 ± 0.17^{abc}	0.06 ± 0.01^{abc}
21	Control_2	0.02 ± 0.00^{c}	$0.02\pm0.00^{k-n}$	0.02 ± 0.00^{f}	$0.09\pm0.00^{e-l}$	$0.17\pm0.01^{b-e}$	$1.89\pm0.02^{c-i}$	$2.09\pm0.05^{a-h}$	$0.75\pm0.05^{d-j}$	0.08 ± 0.33^{b}	0.65 ± 0.09^{abc}	0.09 ± 0.00^{abcc}
	Control_4	0.08 ± 0.07^{c}	0.01 ± 0.01^{n}	0.02 ± 0.00^{f}	0.05 ± 0.00^{jkl}	$0.09\pm0.03^{f-n}$	1.16 ± 0.21^{ghi}	0.72 ± 0.53^{k}	0.30 ± 0.18^{hij}	0.08 ± 0.43^{b}	0.57 ± 0.45^{bc}	0.20 ± 0.15^{a}
	D2_2	0.33 ± 0.04^{bc}	0.02 ± 0.00^{lmn}	0.02 ± 0.00^{f}	$0.14\pm0.01^{c-i}$	$0.15\pm0.02^{b-f}$	$2.03\pm0.31^{a-i}$	$2.00\pm0.18^{a-j}$	$0.70\pm0.21^{d-j}$	2.91 ± 0.04^{abc}	0.70 ± 0.01^{abc}	0.09 ± 0.01^{abc}
	D2_4	0.38 ± 0.01^{bc}	$0.02\pm0.00^{j-n}$	0.01 ± 0.00^{f}	$0.12\pm0.00^{d-k}$	0.21 ± 0.02^{b}	$1.67\pm0.03^{e-i}$	$1.75\pm0.02^{c-k}$	$0.59\pm0.00^{d-j}$	2.52 ± 0.00^{abc}	1.05 ± 0.00^{abc}	0.05 ± 0.00^{abc}
	F1_2	1.36 ± 1.14^{ab}	$0.04\pm0.02^{f-m}$	0.01 ± 0.01^{f}	$0.10\pm0.00^{e-l}$	$0.07\pm0.03^{i-n}$	0.79 ± 0.10^{i}	0.83 ± 0.24^{ijk}	0.25 ± 0.03^{ij}	2.22 ± 1.94^{abc}	1.90 ± 1.14^{a}	0.06 ± 0.00^{abc}
	F1_4	0.24 ± 0.01^{bc}	0.02 ± 0.00^{mn}	0.01 ± 0.00^{f}	0.23 ± 0.01^{abc}	$0.12\pm0.00^{d-l}$	$1.22\pm0.03^{f-i}$	$1.86\pm0.02^{b-k}$	$0.27\pm0.01^{i-j}$	2.40 ± 0.04^{abc}	0.96 ± 0.02^{abc}	0.04 ± 0.00^{bc}
	JL_2	0.32 ± 0.04^{bc}	0.02 ± 0.00^{lmn}	0.02 ± 0.00^{f}	$0.09\pm0.01^{g-l}$	$0.15\pm0.00^{b-f}$	$1.93\pm0.09^{b-i}$	$2.11\pm0.08^{a-h}$	$0.62\pm0.00^{d-j}$	2.55 ± 0.01^{abc}	0.69 ± 0.02^{abc}	0.09 ± 0.00^{abc}
	JL_4	0.16 ± 0.02^{bc}	0.02±0.00j-n	0.02 ± 0.00^{f}	$0.20\pm0.01^{a-d}$	$0.15\pm0.01^{b-f}$	$1.51\pm0.04^{f-i}$	$2.02\pm0.08^{a-i}$	$0.53\pm0.04^{d-j}$	2.44 ± 0.21^{abc}	0.89 ± 0.10^{abc}	0.06 ± 0.01^{abc}
	Mix_2	0.18 ± 0.00^{bc}	$0.03\pm0.00^{i-n}$	0.02 ± 0.00^{f}	0.28 ± 0.03^{a}	0.18 ± 0.02^{bcd}	$2.10\pm0.14^{a-i}$	$2.10\pm0.20^{a-h}$	$0.72\pm0.11^{d-j}$	2.95 ± 0.27^{abc}	0.75 ± 0.08^{abc}	0.09 ± 0.01^{abc}
	Mix_4	0.17 ± 0.01^{bc}	0.02 ± 0.00^{lmn}	0.01 ± 0.01^{f}	$0.17\pm0.01^{b-f}$	0.20 ± 0.02^{bc}	$1.83\pm0.62^{c-i}$	$1.71\pm0.68^{c-k}$	$0.76\pm0.30^{d-i}$	2.02 ± 0.13^{abc}	1.30 ± 0.22^{abc}	0.06 ± 0.01^{abc}

Values are expressed as the mean ± standard deviation (SD). Different superscript letters indicate a significant difference ($P<0.05$).

Supplementary Table S2: Changes in texture of fermented youlk (*n*=3).

Treatment	Fermentation Time (days)	Hardness (gf)	Cohesiveness	Springiness	Chewiness (gf)
Control_2%	0	21279.39±676.10^{Aab}	0.13±0.01^{Aa}	1.00±0.01^{Aa}	2663.97±142.30^{Aab}
	7	17548.32±1823.45^{ABa}	0.13±0.03^{Aa}	1.01±0.02^{Aa}	2377.12±683.37^{Aa}
	14	14455.10±273.27^{Ba}	0.15±0.02^{Aa}	0.98±0.00^{Aa}	2195.09±212.43^{Aa}
	21	12829.13±2871.23^{B}	0.12±0.02^{Ab}	1.01±0.01^{Aa}	1976.86±541.33^{Ab}
Control_4%	0	21199.54±3544.89^{Aab}	0.28±0.08^{Aa}	0.98±0.00^{Aa}	5788.63±584.18^{Aa}
	7	20980.26±226.27^{Aa}	0.14±0.01^{Aa}	1.00±0.00^{Aa}	2923.09±309.11^{ABa}
	14	16744.17±865.56^{Aa}	0.12±0.02^{Aa}	1.01±0.01^{Aa}	2066.27±438.32^{Baa}
	21	10302.99±369.62^{B}	0.17±0.08^{Aab}	0.98±0.00^{Aa}	1776.16±864.78^{Bb}
D2_2%	0	18227.17±2174.08^{ABb}	0.14±0.05^{Aa}	0.99±0.02^{Aa}	2498.04±591.53^{Ab}
	7	16303.70±962.38^{Ba}	0.25±0.04^{Aa}	0.98±0.00^{Aa}	3016.20±873.70^{Aa}
	14	14495.24±260.31^{Ba}	0.15±0.02^{Aa}	1.02±0.00^{Aa}	2203.10±352.94^{Aa}
	21	11201.07±962.38^{B}	0.13±0.02^{Ab}	1.01±0.02^{Aa}	1492.82±318.34^{Ab}
D2_4%	0	24763.22±547.96^{Aa}	0.13±0.02^{Aa}	1.01±0.01^{Aa}	3947.70±580.77^{Aab}
	7	18330.97±267.32^{Ba}	0.22±0.04^{Aa}	0.98±0.00^{Aa}	3385.19±763.73^{Aa}
	14	15707.97±710.44^{BCa}	0.16±0.03^{Aa}	0.98±0.01^{Aa}	2521.32±570.92^{Aa}
	21	12431.02±413.14^{Ca}	0.12±0.02^{Ab}	0.99±0.00^{Aa}	1473.39±290.05^{Ab}
F1_2%	0	18243.28±728.54^{Ab}	0.11±0.06^{Aa}	1.00±0.00^{Aa}	3011.18±1140.27^{Ab}
	7	16789.89±1476.06^{Aba}	0.19±0.09^{Aa}	0.98±0.00^{Aa}	2517.39±1263.41^{Aa}
	14	16224.87±1380.43^{Ba}	0.14±0.00^{Aa}	1.01±0.01^{Aa}	2175.50±268.06^{Aa}
	21	12119.36±625.26^{Ba}	0.17±0.01^{Aab}	0.98±0.01^{Aa}	1977.96±42.26^{Aab}
F1_4%	0	18923.08±557.16^{Ab}	0.18±0.04^{Aa}	0.98±0.00^{Aa}	3398.54±562.70^{Aab}
	7	17213.25±2043.27^{Aba}	0.11±0.07^{Aa}	0.99±0.02^{Aa}	2864.75±899.18^{Aa}
	14	16266.86±1411.24^{ABa}	0.13±0.01^{Aa}	1.00±0.01^{Aa}	2195.04±427.74^{Aa}
	21	13429.87±498.71^{Ba}	0.14±0.03^{Ab}	1.00±0.03^{Aa}	1949.65±464.05^{Ab}
JL_2%	0	19299.18±178.37^{Ab}	0.14±0.07^{Aa}	1.00±0.01^{Aa}	2728.97±1414.62^{Aab}
	7	17095.52±1010.19^{ABa}	0.11±0.02^{Aa}	1.01±0.02^{Aa}	2132.06±469.60^{Aa}
	14	14723.31±1294.64^{ABa}	0.16±0.01^{Aa}	0.98±0.00^{Aa}	1937.01±381.68^{Aa}
	21	13537.11±44.91^{Ba}	0.11±0.05^{Ab}	1.00±0.02^{Aa}	1561.56±738.89^{Ab}
JL_4%	0	20811.35±72.90^{Aab}	0.12±0.02^{Aa}	1.02±0.01^{Aa}	2603.60±412.20^{Ab}
	7	17640.16±1094.98^{ABa}	0.10±0.01^{Aa}	0.98±0.01^{Aa}	2020.90±117.00^{Aa}
	14	15074.79±205.69^{Ba}	0.22±0.10^{Aa}	0.98±0.00^{Aa}	1227.45±1426.68^{Aa}
	21	13601.95±1344.11^{Ba}	0.15±0.09^{Aab}	0.98±0.00^{Aa}	1061.51±1462.53^{Ab}
Mix_2%	0	21740.12±1963.09^{Aab}	0.13±0.01^{Ba}	0.98±0.00^{Aa}	3717.82±10.70^{Aab}
	7	17756.15±1748.62^{ABa}	0.13±0.00^{Ba}	1.00±0.02^{Aa}	2666.68±253.27^{Aa}
	14	18075.44±955.23^{ABa}	0.13±0.03^{Ba}	1.00±0.01^{Aa}	2406.78±655.85^{Aa}
	21	14962.69±1708.67^{Ba}	0.35±0.07^{Aa}	0.98±0.00^{Aa}	1127.31±1551.34^{Aa}
Mix_4%	0	21159.43±1873.30^{Aab}	0.11±0.03^{Aa}	1.01±0.01^{Aa}	2434.25±911.81^{Ab}
	7	16754.19±595.28^{ABa}	0.12±0.08^{Aa}	1.00±0.01^{Aa}	2254.42±1329.95^{Aa}
	14	16315.05±8.14^{ABa}	0.13±0.00^{Aa}	1.00±0.00^{Aa}	2186.09±6.46^{Aa}
	21	14070.19±932.25^{Ba}	0.16±0.07^{Aab}	0.98±0.01^{Aa}	2078.83±819.38^{Aab}

Values are expressed as the mean ± standard deviation (SD). There is a significant difference between the values that do not share a letter within rows ($P<0.05$).
